# TARGETING MITOCHONDRIAL FUNCTION USING NOVEL RHO/MRTF/SRF INHIBITORS

**DOI:** 10.1093/geroni/igad104.3002

**Published:** 2023-12-21

**Authors:** Pankaj Patyal, Xiaomin Zhang, Gohar Azhar, Jeanne Wei

**Affiliations:** University of Arkansas for Medical Sciences, Little Rock, Arkansas, United States; University of Arkansas for Medical Sciences, Little Rock, Arkansas, United States; University of Arkansas for Medical Sciences, Little Rock, Arkansas, United States; University of Arkansas for Medical Sciences, Little Rock, Arkansas, United States

## Abstract

Altered mitochondrial metabolism is one of the major hallmarks of aging. Notably, age is the biggest risk factor for numerous cancers. Studies suggest that cancer cells shift from anaerobic respiration to oxidative phosphorylation for increasing energy needs. Therefore, this upregulation is a vulnerability that can be targeted using Rho/MRTF/SRF inhibitors. Recent advancement identified a novel series of oxadiazole-thioether compounds that disrupt the SRF transcription. Among those compounds CCG-203971 and CCG-232601 are most metabolic stable, soluble and have no toxicity. They have been shown efficacious in multiple animal models of acute fibrosis, including scleroderma. However, the direct molecular target of these compounds is unclear. Therefore, we investigated the molecular mechanisms of these inhibitors transcription mediated by RhoA which modulates actin dynamics to induce the nuclear accumulation of Myocardin-Related Transcription Factors and their activation of SRF/p49. Our findings suggest that, CCG-203971 and CCG-232601, inhibit Rho/MRTF/SRF/p49 mediated transcription in different cell lines. They contribute to H4 hyperacetylation at K12/16 while no change was observed at H4 acetylation at K5 and K8. Moreover, SRF also regulates cell metabolism by maintaining mitochondrial function. Thus, we further investigated the role of these drug molecules in targeting mitochondrial function. We found that these compounds inhibit the genes involved in mitochondrial function and dynamics. Further, these inhibitors repress the mitochondrial function at varying concentrations, investigated using Agilent Seahorse XF Analyzer. Herein, we propose that CCG-203971 and CCG-232601 may prove to be a promising approach therapeutically to target aberrant bioenergetics in aging and cancer.

